# Enzymatic changes in myosin regulatory proteins may explain vasoplegia in terminally ill patients with sepsis

**DOI:** 10.1042/BSR20150207

**Published:** 2016-03-03

**Authors:** Wentao Zheng, Yong Kou, Feng-lan Gao, Xiu-he Ouyang

**Affiliations:** * Incentive Care Unit, Binzhou People Hospital, Shandong, Binzhou 256600, China; † Department of Cardiovascular Medicine, Binzhou People Hospital, Shandong, Binzhou 256600, China; ‡ Department of Nephrology, Binzhou People Hospital, Shandong, Binzhou 256600, China

**Keywords:** cardiocirculatory collapse, myosin light chain kinase (MLCK), pharmacomechanical coupling, sepsis

## Abstract

The current study was conducted with the hypothesis that failure of maintenance of the vascular tone may be central to failure of the peripheral circulation and spiralling down of blood pressure in sepsis. Namely, we examined the balance between expression of myosin light chain (MLC) phosphatase and kinase, enzymes that regulate MLCs dephosphorylation and phosphorylation with a direct effect on pharmacomechanical coupling for smooth muscle relaxation and contraction respectively. Mechanical recordings and enzyme immunoassays of vascular smooth muscle lysates were used as the major methods to examine arterial biopsy samples from terminally ill sepsis patients. The results of the present study provide evidence that genomic alteration of expression of key regulatory proteins in vascular smooth muscles may be responsible for the relentless downhill course in sepsis. Down-regulation of myosin light chain kinase (MLCK) and up-regulation of MLCK may explain the loss of tone and failure to mount contractile response *in vivo* during circulation. The mechanical studies demonstrated the inability of the arteries to develop tone when stimulated by phenylephrine *in vitro*. The results of our study provide indirect hint that control of inflammation is a major therapeutic approach in sepsis, and may facilitate to ameliorate the progressive cardiovascular collapse.

## Abstract (precis)

The results of our study provide indirect hint that control of inflammation is a major therapeutic approach in sepsis, and may facilitate to ameliorate the progressive cardiovascular collapse.

## INTRODUCTION

Sepsis continues to be a devastating condition globally encountered for patients admitted in institutionalized settings [1,2]. Despite aggressive management of infections and maintenance of routine conditions like fluid and electrolyte balance and nutritional support [3], patients continue to succumb to sepsis [4-8]. One of the hallmarks of this continuum of clinical degradation is the inability to sustain the circulatory pressure and increased demands for inotropic support [3,9]. After a stage, even inotropic support with increasing doses fail, leading to progressive cardiovascular collapse [5,10-14].

One of the major mechanisms that operate in the peripheral vascular system to sustain circulation is by maintenance of vascular tone [15-17]. This is performed by a biochemical mechanism of modulating contraction state of myosin and its components, which is thereby transduced to a mechanical state of contraction of the smooth muscles in the wall of the blood vessel [18-21].

In the present study, we hypothesized that failure of maintenance of the vascular tone may be central to failure of the peripheral circulation and spiralling down of blood pressure in sepsis. Namely, we examined the balance between expression of myosin light chain (MLC) phosphatase and kinase, enzymes that regulate MLCs dephosphorylation and phosphorylation with a direct effect on pharmacomechanical coupling for smooth muscle relaxation and contraction respectively [21].

## PATIENTS AND METHODS

### Ethical clarification

Permission was obtained from Institutional Review Board (IRB) of Binzhou People Hospital and all experiments were performed strictly in compliance with Helsinki guidelines for ethical experiments with human tissues.

### Setting and duration of study

Tissues were procured from men between the ages of 63 and 78 years old and were used in the present study.

### Patient population

All patients were admitted in the Intensive Care Unit. Some of the patients had received inotropic support but progressive hypotension was the cause of death. All the patients had advanced sepsis, which were pharmacologically uncontrollable and rapidly progressed to septic shock and demise. Systolic pressures for the assigned patients were always less than 90 mmHg to start with. Explicit consent was obtained from the patient's family members. All patient admission, triaging and acute care, fluid resuscitation and inotropic support management was performed according to the guidelines outlined in the Surviving Sepsis Campaign [22].

### Sample procurement

All arterial biopsy samples were obtained during the post-mortem examination. Artery samples (*n*=10 for each group examined, all the four different categories of arteries dissected from each subject and dissected bilaterally except for the aorta) were stored in gassed Krebs solution that was kept in an ice beaker. Arterial sample pieces from different zones were used for mechanical recordings. Samples were frozen in liquid nitrogen and rethawed under similar conditions to perform the biochemical assays. Normal subjects were ones with mortality unrelated to any sepsis or infectious conditions (mainly, these subjects had long standing cardiovascular risk factors but none of the patients had any episode of myocardial infarction, stroke, thyroid or any other endocrine diseases or immunologic diseases). Because of the issue of age matching, we included the elderly subjects as controls who might have alterations of vascular parameters in comparison with younger controls. But because of the homogeneity of the group age, we considered this as our control group. In future studies, we may include younger subjects under which circumstances, there may be some changes in the baseline parameters.

### Mechanical recordings of responses of arterial mounts to a contracting stimulus

Biopsy samples of arteries were cleaned of adherent fat tissues prior to mounting for mechanical studies. The respective blood vessels in each cohort were harvested and preserved in ice-cold Kreb's buffer (sodium chloride 125 mM; potassium chloride 5.9 mM; sodium dihydrogenphosphate 1 mM; sodium bicarbonate 25 mM; magnesium chloride 1.2 mM; calcium chloride 2.5 mM; dextrose 12 mM) for contractility analyses. The arteries were carefully cut into strips and the strips were suspended in an organ bath maintained at 37°C and bubbled with a 95% O_2_/5% CO_2_. Tissues were connected to a force transducer (Grass Instruments), initially stretched to a resting tension of 1.5 g and equilibrated for an hour. Contractile responses to the adrenergic agent phenylephrine was used to measure contractile responses and confirmed with potassium chloride (120 mM). Signals from force transducers were recorded in real time at 30 Hz by a 16-channel analog-to-digital transducer (DataQ, DI-720) and records were transferred to a disk using Windaq data acquisition software. The force changes were computed as force (micro-Newton) normalized by tissue weight and tabulated as mean±S.E.M. Comparisons was made between the different blood vessels between control patients and patients with sepsis.

### ELISA to detect expression levels of different proteins that maintain vascular smooth muscle tone

Enzyme linked immunoassay was utilized in the present study to identify specific protein expression in arterial tissue lysates obtained from patients with sepsis and healthy controls. These studies were focused on known markers of key regulators of pharmacomechanical coupling that maintain vascular smooth muscle tone. By phosphorylating myosin light chain kinase (MLCK), MLCs associate with myosin heavy chains (MHCs) and thereafter form strong acto-myosin interactions to phasically increase tone in the vascular bed [21]. This is reversed when a phosphatase, MLC dephosphatase helps in uncoupling of acto-myosin interactions, thus relaxing the vascular wall [21]. Readings were obtained in triplicates on a standard plate-reader and values averaged over the readings to obtain the final results. Ten patient samples and equal number of controls were used in the present study and all studies performed in triplicates. ELISA kits were obtained from Novus, Biomatik and Cusabio.

### Statistics

Results of all quantitative estimates were shown as mean±S.E.M. Student's *t* test was used to compare differences of means between groups of arterial samples of different physiological functions (peripheral conductive artery, resistance vessels and vessel with auto-regulatory function). Unpaired *t* test was performed as there was heterogeneity in the age groups included. The datasets were tested for Gaussian distribution prior to analyses.

## RESULTS

### Decrease in agonist-evoked contractions of different arterial strips in samples from patients with sepsis

**Figure 1:**
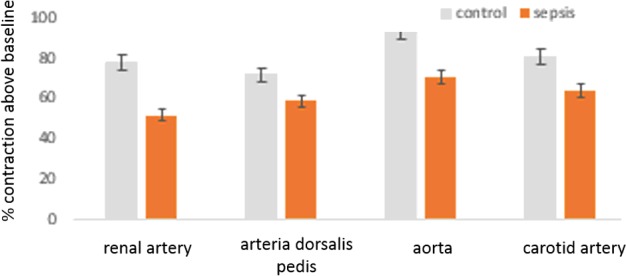
Histograms showing inability of vascular smooth muscles of different arteries to attain agonist-evoked maximal contraction The arteries were obtained from patients with sepsis (*n*=10 subjects) and age-matched controls (*n*=10 subjects) from mortality of non-septic conditions. Cumulative datasets are shown. Means were significantly different when compared between groups. *, significantly decrease.

Renal artery and aorta were examined for major arteries related to resistance development in the vasculature. Carotid artery was tested, as it has its self-auto-regulatory mechanism. Arteria dorsalis pedis was tested as a model for a peripheral artery. In contrast with control subjects, arterial samples obtained from patients with sepsis showed persistent inability to rise to maximal contraction levels with an adrenergic agonist or depolarization by potassium chloride. Cumulative comparative data reflecting this trend in different arterial beds is demonstrated in Figure 1. The means were significantly different between groups (*P*<0.01, *t* test), when different sets of vessels were compared between normal samples and those obtained from patients with advanced sepsis.

### Increase in MLC phosphatase protein expression in arterial lysates of sepsis patients

**Figure 2:**
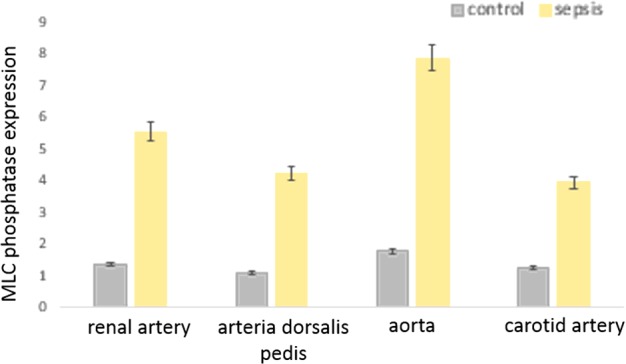
Histograms showing significant increase in expression of MLC phosphatase in vascular smooth muscle lysates obtained from patients with sepsis (*n*=10 in each set of arteries) *, Significantly increased. The representative arteries examined include resistance, conductance and vessels with auto-regulatory functions respectively.

ELISA demonstrated that in all arterial samples examined (resistance vessels, central vessel, peripheral artery), there was persistent severalfolds increase in expression of MLC phosphatase (*P*<0.01, between individual arterial samples, Student's *t* test, each individual group examined respectively) (Figure 2).

### Decrease in MLC kinase protein expression in arterial lysates of sepsis patients

**Figure 3:**
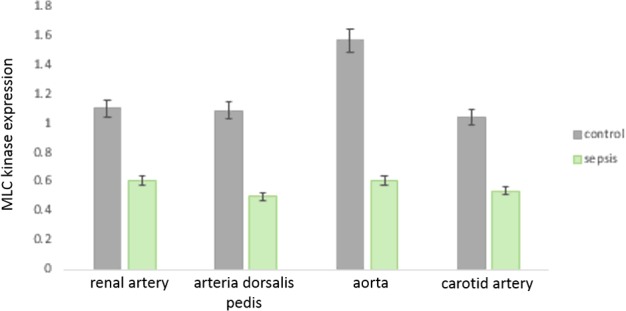
Histograms showing significant decrease in expression of MLCK in vascular smooth muscle lysates obtained from patients with sepsis (*n*=10 in each set of arteries) *, Significantly decreased. The representative arteries examined include resistance, conductance and vessels with auto-regulatory functions respectively.

Enzyme linked assay demonstrated that in all arterial samples examined (resistance vessels, central vessel, peripheral artery), there was persistent decrease in expression of MLCK (*P*<0.01, between individual arterial samples, Student's *t* test, each individual group examined respectively) (Figure 3).

### Myosin heavy and light chain expression unchanged in arterial lysates of sepsis patients

**Figure 4:**
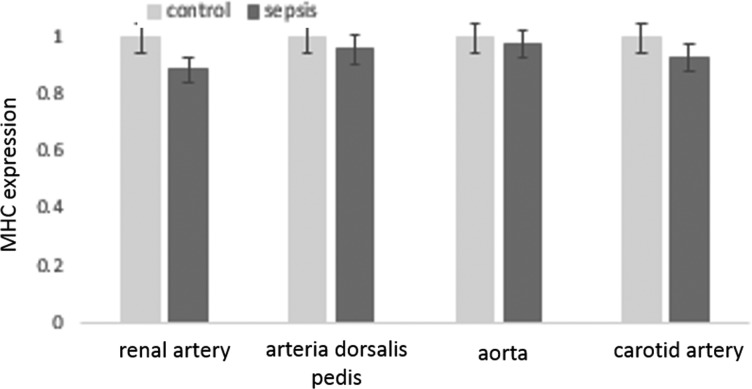
Histograms showing unaltered levels of expression of MHC in vascular smooth muscle lysates obtained from patients with sepsis and control subjects (*n*=10 in each set of arteries) The means were not significantly different between the individual groups of vessels examined.

**Figure 5:**
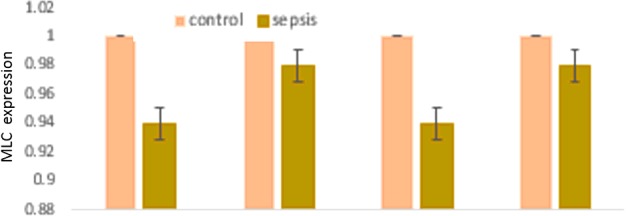
Histograms showing unaltered levels of expression of MLC in vascular smooth muscle lysates obtained from patients with sepsis and control subjects (*n*=10 in each set of arteries) The means were not significantly different between the individual groups of vessels examined.

Protein expressions for smooth muscle specific myosin heavy and light chains were examined, as their changes potentially can affect vascular tone. In all vessels examined, these basal motor molecules that is the major contributor to vascular contractile potential remained unchanged and were nearly similar in expression between control subjects and sepsis patients (Figures 4 and 5).

## DISCUSSION

The results of the present study provide the preliminary evidence that genomic alteration of expression of key regulatory proteins in vascular smooth muscles may be responsible for the relentless downhill course in sepsis. Namely, down-regulation of MLCK, the major enzyme that helps in force generation by aiding association of light chains with the main MHC molecule and facilitates acto-myosin formation [21-24], is a key event that prevents resistance arteries to maintain tone and circulatory blood pressure. Phosphorylation of Ser^19^ on the 20-kDa regulatory light chain (RLC) of myosin II (MLC20) facilitated by Ca^2+^/calmodulin-dependent MLCK is the first and most important step for smooth muscle contraction. These are reversed by activity of the MLC phosphatase.

Associated with these observations were also the fact observed in the present study that MLC phosphatase expression is increased in the different vascular smooth muscles of the arterial biopsies examined. This enzyme dissociates light chains from MHC and causes sustained decrease in vascular smooth muscle tone. These enzymatic changes likely also explain the observations of the mechanical recordings in the present study regarding the inability of the arteries to develop tone when stimulated by phenylephrine *in vitro*. Maximal depolarization with increasing concentrations of potassium chloride also failed to elicit sustained contraction in the arterial samples obtained from the patients with sepsis (results not shown). This provided indirect hint that alteration in the contractile mechanism, rather than effects on the electrogenic potential was responsible for the lack of generation of tone in the arteries in patients with sepsis.

The results of the present study also provided evidence that the quantities of the myosin heavy and light chains remained unaltered. The present study did not perform a time-based examination due to pragmatic reasons of obtaining the biopsies during the acute illnesses of the patients. The observations are endpoint results. However, they provide reasonable basis of the clinical observations of progressive cardiovascular collapse during the terminal stages of the illness.

Recent studies have shown that up-regulation of inducible nitric oxide synthase (iNOS) during uncontrolled infection, inflammation and sepsis have the ability to alter genomic expression of several genes through alteration of nuclear factor κB (NF-κB) signalling [15,25-27]. Some of these genes also participate in pharmacomechanical coupling of smooth muscles [28-29]. The results of our study provide suggestions that these pathways may be linked in progressive pathophysiology of sepsis and that the currently accepted approaches of control of inflammation is rationally beneficial in sepsis, and may facilitate to ameliorate the cardiovascular collapse. However, novel approaches may also be derived to contain relentless sepsis by manipulating expression of MLC regulatory enzyme panels including phosphatases and kinases. These remain the aim of our future studies.

## Abbreviations

MHC: myosin heavy chain
MLC: myosin light chain
MLCK: myosin light chain kinase

## References

[1] Paulsen J., Mehl A., Askim Å., Solligård E., Åsvold B.O., Damås J.K. (2015). Epidemiology and outcome of *Staphylococcus*
*aureus* bloodstream infection and sepsis in a Norwegian county 1996–2011: an observational study. BMC Infect. Dis..

[2] Oud L., Watkins P. (2015). Evolving trends in the epidemiology, resource utilization, and outcomes of pregnancy-associated severe sepsis: a population-based cohort study. J. Clin. Med. Res..

[3] Mouncey P.R., Osborn T.M., Power G.S., Harrison D.A., Sadique M.Z., Grieve R.D., Jahan R., Harvey S.E., Bell D., Bion J.F. (2015). Trial of early, goal-directed resuscitation for septic shock. N. Engl. J. Med..

[4] Ostrowski S.R., Haase N., Müller R.B., Møller M.H., Pott F.C., Perner A., Johansson P.I. (2015). Association between biomarkers of endothelial injury and hypocoagulability in patients with severe sepsis: a prospective study. Crit. Care.

[5] Zhang L., Zhu G., Han L., Fu P. (2015). Early goal-directed therapy in the management of severe sepsis or septic shock in adults: a meta-analysis of randomized controlled trials. BMC Med..

[6] Tagami T., Matsui H., Fushimi K., Yasunaga H. (2015). Intravenous immunoglobulin and mortality in pneumonia patients with septic shock: an observational nationwide study. Clin. Infect. Dis..

[7] Wang H.E., Addis D.R., Donnelly J.P., Shapiro N.I., Griffin R.L., Safford M.M., Baddley J.W. (2015). Discharge diagnoses versus medical record review in the identification of community-acquired sepsis. Crit. Care.

[8] Semler M.W., Weavind L., Hooper M.H., Rice T.W., Gowda S.S., Nadas A., Song Y., Martin J.B., Bernard G.R., Wheeler A.P. (2015). An electronic tool for the evaluation and treatment of sepsis in the ICU: a randomized controlled trial. Crit. Care Med..

[9] Honore P.M., Jamez J., Wauthier M., Lee P.A., Dugernier T., Pirenne B., Hanique G., Matson J.R. (2000). Prospective evaluation of short-term, high-volume isovolemic hemofiltration on the hemodynamic course and outcome in patients with intractable circulatory failure resulting from septic shock. Crit. Care Med..

[10] Hoover D.B., Ozment T.R., Wondergem R., Li C., Williams D.L. (2015). Impaired heart rate regulation and depression of cardiac chronotropic and dromotropic function in polymicrobial sepsis. Shock.

[11] Beck V., Chateau D., Bryson G.L., Pisipati A., Zanotti S., Parrillo J.E., Kumar A., Cooperative Antimicrobial Therapy of Septic Shock (CATSS) Database Research Group (2014). Timing of vasopressor initiation and mortality in septic shock: a cohort study. Crit. Care.

[12] Oba Y., Lone N.A. (2014). Mortality benefit of vasopressor and inotropic agents in septic shock: a Bayesian network meta-analysis of randomized controlled trials. J. Crit. Care.

[13] Ranjit S., Kissoon N. (2013). Bedside echocardiography is useful in assessing children with fluid and inotrope resistant septic shock. Indian J. Crit. Care Med..

[14] Bangash M.N., Kong M.L., Pearse R.M. (2012). Use of inotropes and vasopressor agents in critically ill patients. Br. J. Pharmacol..

[15] Gamkrelidze M., Intskirveli N., Vardosanidze K., Kh Chikhladze, Goliadze L., Ratiani L. (2015). Vasoplegia in septic shock (review). Georgian Med. News.

[16] Guinot P.G., Bernard E., Levrard M., Dupont H., Lorne E. (2015). Dynamic arterial elastance predicts mean arterial pressure decrease associated with decreasing norepinephrine dosage in septic shock. Crit. Care.

[17] Chawla L.S., Ince C., Chappell D., Gan T.J., Kellum J.A., Mythen M., Shaw A.D., ADQI XII Fluids Workgroup (2014). Vascular content, tone, integrity, and haemodynamics for guiding fluid therapy: a conceptual approach. Br. J. Anaesth..

[18] Morgado M., Cairrão E., Santos-Silva A.J., Verde I. (2012). Cyclic nucleotide-dependent relaxation pathways in vascular smooth muscle. Cell. Mol. Life Sci..

[19] Khalil R.A. (2010). Regulation of Vascular Smooth Muscle Function.

[20] Snetkov V.A., Smirnov S.V., Kua J., Aaronson P.I., Ward J.P., Knock G.A. (2011). Superoxide differentially controls pulmonary and systemic vascular tone through multiple signalling pathways. Cardiovasc. Res..

[21] Ogut O., Brozovich F.V. (2003). Regulation of force in vascular smooth muscle. J. Mol. Cell. Cardiol..

[22] Dellinger R.P., Levy M.M., Rhodes A., Annane D., Gerlach H., Opal S.M., Sevransky J.E., Sprung C.L., Douglas I.S., Jaeschke R. (2013). Surviving sepsis campaign: international guidelines for management of severe sepsis and septic shock: 2012. Crit. Care Med..

[23] Somlyo A.V., Somlyo A.P. (1993). Intracellular signaling in vascular smooth muscle. Adv. Exp. Med. Biol..

[24] Somlyo A.V., Somlyo A.P. (1968). Electromechanical and pharmacomechanical coupling in vascular smooth muscle. J. Pharmacol. Exp. Ther..

[25] Reho J.J., Zheng X., Asico L.D., Fisher S.A. (2015). Redox signaling and splicing dependent change in myosin phosphatase underlie early versus late changes in NO vasodilator reserve in a mouse LPS model of sepsis. Am. J. Physiol. Heart Circ. Physiol..

[26] Cubillos-Zapata C., Hernández-Jiménez E., Toledano V., Esteban-Burgos L., Fernández-Ruíz I., Gómez-Piña V., Del Fresno C., Siliceo M., Prieto-Chinchiña P., Pérez de Diego R. (2014). NFκB2/p100 is a key factor for endotoxin tolerance in human monocytes: a demonstration using primary human monocytes from patients with sepsis. J. Immunol..

[27] Schäfer S.T., Gessner S., Scherag A., Rump K., Frey U.H., Siffert W., Westendorf A.M., Steinmann J., Peters J., Adamzik M. (2014). Hydrocortisone fails to abolish NF-κB1 protein nuclear translocation in deletion allele carriers of the NFKB1 promoter polymorphism (-94ins/delATTG) and is associated with increased 30-day mortality in septic shock. PLoS One.

[28] Lamounier-Zepter V., Baltas L.G., Morano I. (2003). Distinct contractile systems for electromechanical and pharmacomechanical coupling in smooth muscle. Adv. Exp. Med. Biol..

[29] Löhn M., Kämpf D., Gui-Xuan C., Haller H., Luft F.C., Gollasch M. (2002). Regulation of arterial tone by smooth muscle myosin type II. Am. J. Physiol. Cell Physiol..

